# Association of Late Preterm Birth and Size for Gestational Age With Cardiometabolic Risk in Childhood

**DOI:** 10.1001/jamanetworkopen.2022.14379

**Published:** 2022-05-27

**Authors:** Yulika Yoshida-Montezuma, Branavan Sivapathasundaram, Hilary K. Brown, Charles Keown-Stoneman, Russell J. de Souza, Teresa To, Cornelia M. Borkhoff, Catherine S. Birken, Jonathon L. Maguire, Laura N. Anderson

**Affiliations:** 1 Department of Health Research Methods, Evidence, and Impact, McMaster University, Hamilton, Ontario, Canada; 2 ICES (formerly the Institute for Clinical Evaluative Sciences), Sunnybrook Health Sciences Centre, Toronto, Ontario, Canada; 3 Dalla Lana School of Public Health, University of Toronto, Toronto, Ontario, Canada; 4 Department of Health and Society, University of Toronto Scarborough, Toronto, Ontario, Canada; 5 Women’s College Research Institute, Toronto, Ontario, Canada; 6 Li Ka Shing Knowledge Institute, St Michael’s Hospital, Toronto, Ontario, Canada; 7 Department of Nutritional Sciences, Faculty of Medicine, University of Toronto, Toronto, Ontario, Canada; 8 Population Health Research Institute, Hamilton Health Sciences Corporation, Hamilton, Ontario, Canada; 9 Child Health Evaluative Sciences, The Hospital for Sick Children Research Institute, Toronto, Ontario, Canada; 10 Division of Pediatric Medicine, The Hospital for Sick Children, Toronto, Ontario, Canada; 11 Institute of Health Policy, Management and Evaluation, Dalla Lana School of Public Health, University of Toronto, Toronto, Ontario, Canada; 12 Department of Pediatrics, Faculty of Medicine, University of Toronto, Toronto, Ontario, Canada; 13 Department of Pediatrics, St Michael’s Hospital, Unity Health Toronto, Toronto, Ontario, Canada

## Abstract

**Importance:**

The long-term cardiometabolic consequences of late preterm birth (34-36 weeks’ gestation) are not well understood.

**Objective:**

To assess whether late preterm birth and size for gestational age are associated with cardiometabolic risk (CMR) in childhood.

**Design, Setting, and Participants:**

This retrospective cohort study included 1742 children born in Ontario, Canada, between April 1, 2006, and September 30, 2014, and followed up until September 30, 2019. Data from children enrolled in The Applied Research Group for Kids (TARGet Kids!) primary care practice–based research network were linked to administrative health care data at ICES (formerly known as the Institute for Clinical Evaluative Sciences). Participants were excluded if they had conditions affecting growth (eg, failure to thrive or cystic fibrosis), any acute or chronic conditions (other than asthma and high-functioning autism), severe developmental delay, or families who were unable to communicate in English.

**Exposures:**

Late preterm birth, gestational age as a continuous measure, and size for gestational age.

**Main Outcomes and Measures:**

The primary outcome was composite CMR score (overall age- and sex-standardized *z* score of CMR components, including waist circumference, log triglyceride level, glucose level, systolic blood pressure, and high-density lipoprotein cholesterol level). Secondary outcomes were the individual CMR components. Multivariable linear regression analysis was used to separately evaluate the associations of late preterm birth, continuous gestational age, and size for gestational age with CMR at ages 3 to 12 years.

**Results:**

Among 2440 eligible children, 1742 (mean [SD] age, 5.6 [2.2] years; 951 boys [54.6%]) were included in the final cohort. Overall, 87 children (5.0%) were born moderately preterm (<34 weeks’ gestation), 145 (8.3%) were born late preterm (34-36 weeks’ gestation), 455 (26.1%) were born early term (37-38 weeks’ gestation), and 1055 (60.6%) were born full term (≥39 weeks’ gestation). Compared with children born full term, those born moderately preterm (adjusted β = 0.50; 95% CI, 0.24-0.75) and late preterm (adjusted β = 0.27; 95% CI, 0.06-0.47) had higher CMR scores. Each additional gestational week was associated with a 0.06 U (adjusted β; 95% CI, –0.08 to –0.03 U) decrease in CMR.

**Conclusions and Relevance:**

In this study, children born late preterm and moderately preterm had higher CMR. These results suggest that screening and early-life interventions for these children may prevent cardiometabolic outcomes.

## Key Points

**Question:**

Are late preterm birth and size for gestational age associated with cardiometabolic risk (CMR) in childhood?

**Findings:**

In this cohort study including 1742 children aged 3 to 12 years, those born late preterm and moderately preterm had significantly higher CMR compared with those born full term.

**Meaning:**

This study’s findings suggest that because the CMR score tracks risk from childhood into adulthood, late preterm and moderately preterm birth may be important risk factors for cardiometabolic disorders later in life.

## Teaser

This cohort study assesses whether late preterm birth and size for gestational age are associated with cardiometabolic risk in childhood among participants aged 3 to 12 years.

## Introduction

It is well established that early-life exposures during the intrauterine and early postnatal period are associated with risk of adult-onset chronic diseases.^1^ However, most of the evidence is limited to outcomes in adults born extremely preterm (<28 weeks’ gestation) or very preterm (<32 weeks’ gestation). Late preterm birth (34-36 weeks’ gestation) now accounts for 75% of all preterm births, and this proportion has been steadily increasing since 1990.^2^ Increases in obstetrical interventions, such as induced and cesarean delivery, account for part of the increase in late preterm births, but much of the reason for this increase remains unexplained.^3,4,5^ In addition, risk factors for spontaneous preterm delivery, including older maternal age, multiple-gestation births, maternal obesity, and maternal diabetes, have also been increasing.^6,7^ There is growing awareness that late preterm newborns are not as physiologically or metabolically mature as term newborns and that late preterm birth may confer negative consequences across a child’s life course.^8^ Size for gestational age, a measure that incorporates infant size at birth, has also been acknowledged as having an impact for health later in life through altered childhood growth and overweight status.^9,10^

A systematic review^11^ examining the association between late preterm birth and cardiometabolic health outcomes suggested that children younger than 18 years who were born late preterm vs term were at increased risk of diabetes (pooled adjusted risk ratio from 9 studies: 1.24 [95% CI, 1.17-1.32]) and hypertension (pooled adjusted risk ratio from 11 studies: 1.21 [95% CI, 1.13-1.30]). Studies^12,13^ have also found that adults born late preterm compared with term had high levels of cardiometabolic risk (CMR) factors, including higher body fat percentage, higher blood pressure, metabolic syndrome, and stroke. Small for gestational age (SGA) status has been associated with cardiometabolic outcomes in adulthood,^14^ and children born SGA and large for gestational age (LGA) are at increased risk of overweight and obesity in childhood and adulthood.^10^ It is well established that CMR factors do not occur in isolation,^15^ and the use of a continuous CMR score may better capture potential disease risk. However, the association between CMR score and late preterm birth has not yet been investigated. Among children, CMR is commonly defined as a continuous risk score based on the clustering of age- and sex-standardized central adiposity, high serum lipid levels, glucose levels, and blood pressure.^16^ The objectives of this cohort study were to evaluate whether late preterm birth, gestational age as a continuous measure, and size for gestational age were associated with CMR among children aged 3 to 12 years who were participating in a cohort study in Toronto, Canada.

## Methods

### Study Design

We conducted a retrospective cohort study of children participating in The Applied Research Group for Kids (TARGet Kids!) primary care practice–based research network in Toronto, Canada. Data from children in TARGet Kids! who had CMR outcomes measured at ages 3 to 12 years between April 1, 2006, and September 30, 2019, were individually linked to health care administrative databases at ICES (formerly known as the Institute for Clinical Evaluative Sciences) in Ontario, Canada. Ontario has a universal health care system through which care is provided to almost all residents. ICES is an independent nonprofit research institute whose legal status under section 45 of the Ontario Personal Health Information Protection Act allows it to collect and analyze health care and demographic data without requiring individual participant consent for health system evaluation and improvement. Ethics approval was granted by the Hospital for Sick Children, Unity Health Toronto, and the Hamilton Integrated Research Ethics Boards. All parents of TARGet Kids! participants provided written consent for their child’s data to be linked to health administrative data. This study followed the Strengthening the Reporting of Observational Studies in Epidemiology (STROBE) reporting guideline for cohort studies.

### Study Population and Data Sources

#### Study Population

TARGet Kids! is an open longitudinal cohort study established in 2008.^17^ Children younger than 6 years at enrollment were recruited from pediatric or family practice clinics primarily in the greater Toronto area, where questionnaires and physical measures are collected at regularly scheduled health supervision visits.^17^ Methods used in the TARGet Kids! study have been described previously.^18^ Children were excluded at enrollment if they had health conditions affecting growth (eg, failure to thrive or cystic fibrosis), any acute or chronic conditions (other than asthma and high-functioning autism), severe developmental delay, or families who were unable to communicate in English.^17^ Birth at less than 32 weeks’ gestational age (considered very preterm) was a TARGet Kids! exclusion criterion at enrollment. However, some parents or caregivers did not know their child’s gestational age, and these children were included in the analysis; thus, a small number of children with gestational age less than 32 weeks were included. The flowchart of study participants is provided in the Figure.

**Figure.**
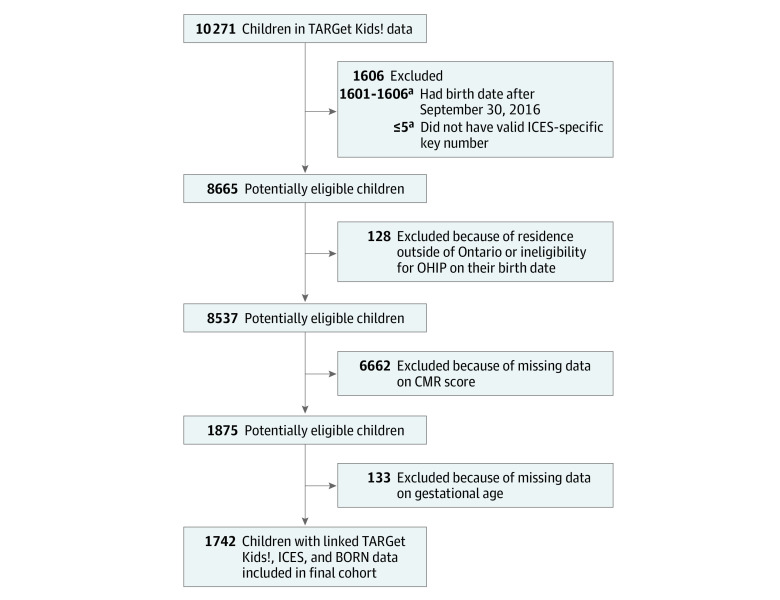
Flowchart of Study Participants BORN indicates Better Outcomes Registry and Network; CMR, cardiometabolic risk; OHIP, Ontario Health Insurance Plan; and TARGet Kids!, The Applied Research Group for Kids primary care practice–based research network. ^a^Exact numbers are not provided to suppress small counts and reduce the possibility of disclosing identifiable data.

#### Data Sources

Data from TARGet Kids! were linked (with 98% success) with the ICES Mother-Baby database,^19^ a maternal-newborn data set that identifies hospital discharge records from the Canadian Institute for Health Information Discharge Abstract Database.^20^ The ICES databases include perinatal data for children born between April 1, 2006, and September 30, 2014, with linkages to maternal data. Those data were also linked to the Better Outcomes Registry and Network (BORN) using unique encoded identifiers; BORN is a provincewide registry of all births in Ontario, Canada, and is part of the Children’s Hospital of Eastern Ontario. Data in this registry are routinely collected from medical records, clinical forms, and patient interviews.^21^ A validation study found greater than 90% agreement between BORN and patient medical records for most of the audited variables.^21^ Data from BORN were used to identify home births and measure maternal body mass index (BMI; calculated as weight in kilograms divided by height in meters squared) and pregnancy complications among all participants.

The cohort was further linked with the Canadian Institute for Health Information Discharge Abstract Database to measure hospital admissions and with the Ontario Health Insurance Plan database to measure outpatient visits.^22^ Several disease registries, including the Ontario Diabetes Database (which includes patients with physician-diagnosed diabetes) and the Ontario Hypertension Database (which includes patients with physician-diagnosed hypertension),^22^ which were derived from the health administrative data sets described in the previous paragraphs, were used to measure chronic conditions among mothers.

### Exposures

The primary exposure was gestational age at birth, ascertained from the Mother-Baby database and supplemented with data from BORN to capture home births. Gestational age at birth was calculated based on the best clinical estimate of gestation, including estimates from both ultrasonography and last menstrual period.^23^ Gestational age was analyzed as a categorical variable with 4 groups defined a priori consistent with clinical definitions: moderately preterm (<34 weeks’ gestation), late preterm (34-36 weeks’ gestation), early term (37-38 weeks’ gestation), and full term (≥39 weeks’ gestation, used as the reference group).^24,25^ Secondary analyses evaluated gestational age as a continuous variable. Size for gestational age was investigated using established cutoffs because of the documented U-shaped association between size for gestational age and cardiometabolic disease risk.^10^ Size for gestational age was derived by applying the 1999 reference birth weight curves for the Canadian population to the present study population.^26^ Size for gestational age was analyzed categorically using the following 3 groups: SGA (<10th percentile), appropriate for gestational age (AGA; 10th-90th percentile), and LGA (>90th percentile).

### Outcomes

The primary outcome was CMR score at ages 3 to 12 years identified from TARGet Kids! data. The individual CMR components (waist circumference, log triglyceride level, glucose level, systolic blood pressure [SBP], and high-density lipoprotein [HDL] cholesterol level) were analyzed as secondary outcomes. The overall CMR score was calculated as the total age- and sex-standardized *z* score of these components by summing waist circumference, log triglycerides, glucose, and SBP and subtracting HDL cholesterol, then dividing this value by the square root of 5.^15,27^ The latest (oldest age) measure of CMR was recorded for children with multiple measurements. The distribution of CMR scores is provided in eFigure 1 in the Supplement.

The CMR score was only calculated for children with data available for all components. This score was analyzed continuously, and the associations between the exposures and each of the CMR components were also assessed individually. Nonfasting blood samples, child’s waist circumference, and SBP were measured by trained research staff members using standardized instruments.^28^

### Confounding Variables

Confounding variables were selected a priori based on previous literature^11^ and conceptualized using a directed acyclic graph (eFigure 2 in the Supplement) that included maternal, perinatal, child, and sociodemographic characteristics hypothesized to be associated with both gestational age at birth and child’s CMR but not on the causal pathway. Confounding variables were maternal age at birth, ethnicity, prepregnancy BMI, prepregnancy diabetes, prepregnancy hypertension, gestational diabetes, gestational hypertension, preeclampsia or eclampsia, venous thromboembolism, annual family income, and family history of cardiometabolic conditions or risk factors. Race was not included as a confounding variable because it was not asked in the TARGet Kids! questionnaire, and race-based data had not been collected by ICES at the current time. We also adjusted for child’s age (continuous in years) and sex (female or male) when the outcome was measured. Data sources and operationalization of variables are provided in the eTable in the Supplement.

### Statistical Analysis

Multivariable linear regression analysis was used to estimate β coefficients and 95% CIs for the associations of gestational age at birth with overall CMR and CMR components, which were analyzed individually. The a priori level of significance was 2-tailed *P* = .05. As a secondary analysis, the associations of size for gestational age with overall CMR and CMR components were examined. All analyses were conducted using models that were minimally adjusted (model 1) and fully adjusted (model 2) to assess how the confounding variables changed the observed effect estimates. Model 1 was adjusted for maternal age at delivery, maternal ethnicity, child’s age when the outcome was measured, child’s sex, and annual family income. Model 2 was adjusted for all confounding variables in model 1 plus maternal prepregnancy BMI, maternal prepregnancy cardiometabolic conditions (diabetes and hypertension), gestational CMR (hypertension, diabetes, preeclampsia or eclampsia, and venous thromboembolism), and family history of cardiometabolic conditions. When analyzing individual CMR components, we also controlled for child’s fasting time (time since last meal or snack) at blood sample collection when conducting the multivariable linear regression analysis for the individual outcome of glucose level and controlled for child’s height for the individual outcome of SBP.^28^

Missing data for the covariates were considered missing at random and imputed using multiple imputation with a total of 10 imputations using the PROC MI procedure in SAS software, version 9.4 (SAS Institute Inc). Continuous variables were imputed via predictive mean matching using 100 iterations. Categorical variables were imputed using the Markov chain Monte Carlo method, which assumes that all variables in the imputation model have a joint multivariable normal distribution. There was less than 15% missingness for each variable before imputation (Table 1). Consistent with ICES privacy requirements, ranges were reported for variables applying to 5 or fewer participants to reduce the possibility of disclosing identifiable data. All analyses were conducted at ICES using SAS software, version 9.4.

**Table 1.** Characteristics of Study Participants by Gestational Age at Birth

Characteristic	Births, No. (%)^a^				
	Overall	Moderately preterm (<34 weeks)	Late preterm (34-36 weeks)	Early term (37-38 weeks)	Full term (≥39 weeks)
Total participants, No.	1742	87	145	455	1055
**Maternal**					
Age, mean (SD), y	33.6 (4.5)	34.4 (4.5)	34.2 (4.9)	33.6 (4.7)	33.5 (4.4)
Missing	26 (1.5)	1-5 (1.1-5.7)	1-5 (0.7-3.4)	8 (1.8)	14 (1.3)
Prepregnancy BMI, mean (SD)	23.7 (4.6)	24.3 (6.1)	23.6 (5.3)	23.7 (4.6)	23.7 (4.4)
Missing	253 (14.5)	16 (18.4)	29 (20.0)	71 (15.6)	137 (13.0)
Ethnicity					
African, Arab, Latin American, or multiple ethnicities	238 (13.7)	15 (17.2)	20 (13.8)	59 (13.0)	144 (13.6)
East Asian, South Asian, or Southeast Asian	315 (18.1)	16 (18.4)	31 (21.4)	100 (22.0)	168 (15.9)
European	1028 (59.0)	48 (55.2)	80 (55.2)	253 (55.6)	647 (61.3)
Missing	161 (9.2)	8 (9.2)	14 (9.7)	43 (9.5)	96 (9.1)
Prepregnancy diabetes					
Yes	11 (0.6)	1-5 (1.1-5.7)	1-5 (0.7-3.4)	6 (1.3)	1-5 (0.1-0.5)
No	1731 (99.4)	82-86 (94.3-98.9)	140-144 (96.6-99.3)	449 (98.7)	1050-1054 (99.5-99.9)
Prepregnancy hypertension					
Yes	32 (1.8)	1-5 (1.1-5.7)	1-5 (0.7-3.4)	8 (1.8)	17 (1.6)
No	1710 (98.2)	82-86 (94.3-98.9)	140-144 (96.6-99.3)	447 (98.2)	1038 (98.4)
**Perinatal**					
Gestational diabetes					
Yes	64 (3.7)	3-7 (3.4-8.0)	7-11 (4.8-7.6)	21 (4.6)	30 (2.8)
No	1615 (92.7)	78 (89.7)	132 (91.0)	413 (90.8)	997 (94.5)
Missing	63 (3.6)	1-5 (1.1-5.7)	2-6 (1.4-4.1)	19 (4.2)	35 (3.3)
Gestational hypertension					
Yes	58 (3.3)	1-5 (1.1-5.7)	7-11 (4.8-7.6)	23 (5.1)	23 (2.2)
No	1621 (93.1)	79 (90.8)	132 (91.0)	413 (90.8)	997 (94.5)
Missing	63 (3.6)	3-7 (3.4-8.0)	2-6 (1.4-4.1)	19 (4.2)	35 (3.3)
Preeclampsia or eclampsia					
Yes	34 (2.0)	1-5 (1.1-5.7)	10-14 (6.9-9.7)	15 (3.3)	1-5 (0.1-0.5)
No	1645 (94.4)	79 (90.8)	129 (89.0)	421 (92.5)	1016 (96.3)
Missing	63 (3.6)	3-7 (3.4-8.0)	2-6 (1.4-4.1)	19 (4.2)	34-38 (3.2-3.6)
Venous thromboembolism					
Yes	8 (0.5)	1-5 (1.1-5.7)	0	1-5 (0.2-1.1)	3-7 (0.3-0.7
No	1716 (98.5)	82-86 (94.3-98.9)	140-144 (96.6-99.3)	447 (98.2)	1041 (98.7)
Missing	18 (1.0)	1-5 (1.1-5.7)	1-5 (0.7-3.4)	3-7 (0.7-1.5)	8 (0.8)
**Child**					
Age at outcome, mean (SD), y	5.6 (2.2)	5.9 (2.3)	5.6 (2.2)	5.5 (2.2)	5.6 (2.2)
Sex					
Female	791 (45.4)	39 (44.8)	59 (40.7)	203 (44.6)	490 (46.4)
Male	951 (54.6)	48 (55.2)	86 (59.3)	252 (55.4)	565 (53.6)
CMR *z* score, mean (SD)	–0.03 (1.20)	0.40 (1.40)	0.20 (1.20)	–0.09 (1.10)	–0.07 (1.10)
**Sociodemographic**					
Annual family income, $					
<50 000	145 (8.3)	16 (18.4)	21 (14.5)	42 (9.2)	66 (6.3)
50 000-99 999	480 (27.6)	21 (24.1)	39 (26.9)	131 (28.8)	289 (27.4)
100 000-149 999	120 (6.9)	1-5 (1.1-5.7)	7-11 (4.8-7.6)	35 (7.7)	73 (6.9)
≥150 000	874 (50.2)	44 (50.6)	64 (44.1)	214 (47.0)	552 (52.3)
Missing	123 (7.1)	1-5 (1.1-5.7)	10-14 (6.9-9.7)	33 (7.3)	75 (7.1)
Family history of cardiometabolic conditions					
Yes	517 (29.7)	29-33 (33.3-37.9)	55-59 (37.9-40.7)	142 (31.2)	285 (27.0)
No	1184 (68.0)	53 (60.9)	85 (58.6)	300 (65.9)	746 (70.7)
Missing	41 (2.4)	1-5 (1.1-5.7)	1-5 (0.7-3.4)	13 (2.9)	24 (2.3)

Abbreviations: BMI, body mass index (calculated as weight in kilograms divided by height in meters squared); CMR, cardiometabolic risk.

^a^ Consistent with ICES privacy requirements, ranges were provided to suppress small cell sizes and reduce the possibility of disclosing identifiable data. Percentages may not total 100% because of rounding.

## Results

Among 2440 eligible children, 1742 (mean [SD] age at outcome, 5.6 [2.2] years; 951 boys [54.6%] and 791 girls [45.4%]) were included in the final cohort (Table 1; Figure) . Overall, 87 children (5.0%) were born moderately preterm, 145 (8.3%) were born late preterm, 455 (26.1%) were born early term, and 1055 (60.6%) were born full term. The mean (SD) overall CMR *z* score for the total cohort was −0.03 (1.20). Among mothers, the mean (SD) age was 33.6 (4.5) years. A total of 238 mothers (13.7%) were of African, Arab, Latin American, or multiple ethnicity; 315 (18.1%) were of East Asian, South Asian, or Southeast Asian ethnicity; 1028 (59.0%) were of European ethnicity; and 161 (9.2%) were of unknown ethnicity (missing data).

Children born late preterm vs full term were more likely to be male (86 of 145 children [59.3%] vs 565 of 1055 children [53.6%]) and have lower annual family income (<$50 000: 21 of 145 children [14.5%] vs 66 of 1055 children [6.3%]); their mothers were more likely to be older (mean [SD], 34.2 [4.9] years vs 33.5 [4.4] years) and of non-European ethnicity (51 of 131 mothers [38.9%] vs 312 of 959 mothers [32.5%]) and to have gestational diabetes (7-11 mothers [4.8%-7.6%] vs 30 mothers [2.8%]), gestational hypertension (7-11 mothers [4.8%-7.6%] vs 23 mothers [2.2%]), or preeclampsia or eclampsia (10-14 mothers [6.9%-9.7%] vs 1-5 mothers [0.1%-0.5%]). The percentage of missing values across the covariates ranged from 0% (maternal prepregnancy cardiometabolic conditions and child’s age and sex) to 14.5% (maternal prepregnancy BMI). Additional characteristics of the study population, including CMR outcome score by gestational age categories, are shown in Table 1.

The minimally adjusted (model 1) and fully adjusted (model 2) associations between gestational age and CMR are shown in Table 2. Based on the fully adjusted model, late preterm birth compared with full-term birth was associated with a 0.27 U (adjusted β; 95% CI, 0.06-0.47 U) higher mean overall CMR. Moderately preterm birth was associated with a 0.50 U (adjusted β; 95% CI, 0.24-0.75 U) higher mean overall CMR. When gestational age was evaluated as a continuous variable, each additional gestational week was associated with a –0.06 U (adjusted β; 95% CI, –0.08 to –0.03 U) lower mean overall CMR.

**Table 2.** Association Between Gestational Age and *z* Score–Transformed Overall CMR and Components of CMR Among Children Aged 3 to 12 Years^a^

Variable^b^	Adjusted β (95% CI)	
	Model 1^c^	Model 2^d^
Overall CMR		
Moderately preterm	0.52 (0.26 to 0.77)	0.50 (0.24 to 0.75)
Late preterm	0.29 (0.09 to 0.49)	0.27 (0.06 to 0.47)
Early term	0 (–0.12 to 0.13)	–0.02 (–0.14 to 0.11)
Full term	1 [Reference]	1 [Reference]
Per additional gestational wk	–0.06 (–0.08 to –0.04)	–0.06 (–0.08 to –0.03)
Components of CMR		
Waist circumference		
Moderately preterm	–0.30 (–1.43 to 0.83)	–0.50 (–1.63 to 0.63)
Late preterm	–0.29 (–1.19 to 0.61)	–0.43 (–1.33 to 0.47)
Early term	–0.03 (–0.59 to 0.54)	–0.14 (–0.71 to 0.43)
Full term	1 [Reference]	1 [Reference]
Per additional gestational wk	0.03 (–0.07 to 0.14)	0.07 (–0.04 to 0.17)
Systolic blood pressure^e^		
Moderately preterm	0.67 (0.45 to 0.89)	0.68 (0.46 to 0.90)
Late preterm	0.25 (0.08 to 0.42)	0.25 (0.07 to 0.43)
Early term	–0.02 (–0.13 to 0.09)	–0.02 (–0.13 to 0.09)
Full term	1 [Reference]	1 [Reference]
Per additional gestational wk	–0.06 (–0.08 to –0.04)	–0.06 (–0.08 to –0.04)
Log triglyceride level		
Moderately preterm	0.30 (0.08 to 0.52)	0.29 (0.07 to 0.51)
Late preterm	0.12 (–0.06 to 0.29)	0.09 (–0.09 to 0.27)
Early term	0.06 (–0.05 to 0.17)	0.04 (–0.07 to 0.15)
Full term	1 [Reference]	1 [Reference]
Per additional gestational wk	–0.03 (–0.05 to –0.01)	–0.03 (–0.05 to –0.01)
HDL cholesterol level		
Moderately preterm	–0.26 (–0.48 to –0.04)	–0.27 (–0.50 to –0.05)
Late preterm	–0.26 (–0.44 to –0.09)	–0.28 (–0.46 to –0.10)
Early term	0.05 (–0.06 to 0.16)	0.04 (–0.07 to 0.15)
Full term	1 [Reference]	1 [Reference]
Per additional gestational wk	0.03 (0.01 to 0.05)	0.03 (0.01 to 0.05)
Glucose level^f^		
Moderately preterm	0.14 (–0.07 to 0.35)	0.13 (–0.08 to 0.34)
Late preterm	0.09 (–0.08 to 0.26)	0.07 (–0.10 to 0.24)
Early term	0.09 (–0.02 to 0.19)	0.08 (–0.03 to 0.18)
Full term	1 [Reference]	1 [Reference]
Per additional gestational wk	–0.02 (–0.04 to 0)	–0.02 (–0.04 to 0)

Abbreviations: CMR, cardiometabolic risk; HDL, high-density lipoprotein.

^a^ Estimates were derived using multivariable linear regression analysis among 1742 participants.

^b^ Moderately preterm was defined as birth occurring earlier than 34 weeks’ gestation; late preterm, birth occurring at 34 to 36 weeks’ gestation; early term, birth occurring at 37 to 38 weeks’ gestation; and full term, birth occurring at 39 weeks’ gestation or later.

^c^ Model 1 was adjusted for maternal age, maternal ethnicity, child’s sex, child’s age at outcome, and annual family income.

^d^ Model 2 was adjusted for model 1 variables plus maternal prepregnancy body mass index (calculated as weight in kilograms divided by height in meters squared), maternal prepregnancy diabetes, maternal prepregnancy hypertension, gestational diabetes, gestational hypertension, venous thromboembolism, preeclampsia or eclampsia, and family history of cardiometabolic conditions.

^e^ Systolic blood pressure was adjusted for child’s height.

^f^ Glucose level was adjusted for fasting time at blood sample collection.

For the individual CMR components, compared with full-term birth, late preterm birth (adjusted β = 0.25; 95% CI, 0.07-0.43) and moderately preterm birth (adjusted β = 0.68; 95% CI, 0.46-0.90) were associated with higher SBP based on the fully adjusted model (Table 2). Compared with full-term birth, late preterm birth (adjusted β = –0.28; 95% CI, –0.46 to –0.10) and moderately preterm birth (adjusted β = –0.27; 95% CI, –0.50 to –0.05) were associated with lower HDL cholesterol levels. Only moderately preterm birth was associated with higher triglyceride levels (adjusted β = 0.29; 95% CI, 0.07-0.51) compared with full-term birth. There was no evidence of an association between early-term birth and any of the individual CMR components.

The secondary analysis evaluating the association between size for gestational age and CMR revealed no association between SGA status and overall CMR (adjusted β = –0.09; 95% CI, –0.28 to 0.10) in the fully adjusted model. No association was found between LGA status and overall CMR (adjusted β = 0.16; 95% CI, –0.03 to 0.35) compared with AGA status (Table 3). For the individual CMR components, compared with AGA status, SGA status was associated with higher SBP (adjusted β = 0.21; 95% CI, 0.04-0.38), but LGA status was not (adjusted β = 0.10; 95% CI, –0.07 to 0.26). An inverse association was observed between LGA status and triglyceride levels (adjusted β = –0.20; 95% CI, –0.36 to –0.03) compared with AGA status. Similar results were observed for SGA status and triglyceride levels (adjusted β = –0.12; 95% CI, –0.28 to 0.05); however, these findings were not statistically significant. Being born SGA was associated with a smaller waist circumference (adjusted β = –1.02; 95% CI, –1.86 to –0.18), whereas being born LGA was associated with a larger waist circumference (adjusted β = 1.06; 95% CI, 0.23-1.89) compared with being born AGA.

**Table 3.** Association Between Size for Gestational Age and *z* Score–Transformed Overall CMR and Components of CMR Among Children Aged 3 to 12 Years^a^

Variable	Adjusted β (95% CI)	
	Model 1^b^	Model 2^c^
Overall CMR		
SGA	–0.08 (–0.27 to 0.11)	–0.09 (–0.28 to 0.10)
AGA	1 [Reference]	1 [Reference]
LGA	0.18 (0 to 0.37)	0.16 (–0.03 to 0.35)
Components of CMR		
Waist circumference		
SGA	–0.98 (–1.82 to –0.14)	–1.02 (–1.86 to –0.18)
AGA	1 [Reference]	1 [Reference]
LGA	1.23 (0.40 to 2.07)	1.06 (0.23 to 1.89)
Systolic blood pressure^d^		
SGA	0.21 (0.04 to 0.38)	0.21 (0.04 to 0.38)
AGA	1 [Reference]	1 [Reference]
LGA	0.11 (–0.06 to 0.27)	0.10 (–0.07 to 0.26)
Log triglyceride level		
SGA	–0.11 (–0.27 to 0.06)	–0.12 (–0.28 to 0.05)
AGA	1 [Reference]	1 [Reference]
LGA	–0.19 (–0.36 to –0.03)	–0.20 (–0.36 to –0.03)
HDL cholesterol level		
SGA	–0.03 (–0.19 to 0.14)	–0.03 (–0.20 to 0.14)
AGA	1 [Reference]	1 [Reference]
LGA	0.05 (–0.11 to 0.21)	0.05 (–0.12 to 0.21)
Glucose level^e^		
SGA	0.01 (–0.15 to 0.17)	0 (–0.16 to 0.16)
AGA	1 [Reference]	1 [Reference]
LGA	–0.02 (–0.18 to 0.14)	–0.02 (–0.18 to 0.14)

Abbreviations: AGA, appropriate for gestational age (10th-90th percentile); CMR, cardiometabolic risk; HDL, high-density lipoprotein; LGA, large for gestational age (>90th percentile); SGA, small for gestational age (<10th percentile).

^a^ Estimates were derived using multivariable linear regression analysis among 1742 participants.

^b^ Model 1 was adjusted for maternal age, maternal ethnicity, child’s sex, child’s age at outcome, and annual family income.

^c^ Model 2 was adjusted for model 1 variables plus maternal prepregnancy body mass index (calculated as weight in kilograms divided by height in meters squared), maternal prepregnancy diabetes, maternal prepregnancy hypertension, gestational diabetes, gestational hypertension, venous thromboembolism, preeclampsia or eclampsia, and family history of cardiometabolic conditions.

^d^ Systolic blood pressure was adjusted for child’s height.

^e^ Glucose level was adjusted for fasting time at blood sample collection.

## Discussion

In this retrospective cohort study, late preterm birth was associated with higher CMR scores among children aged 3 to 12 years. Consistent with previous literature,^12,13^ earlier gestational ages were associated with adverse cardiovascular outcomes. Both late and moderately preterm birth were associated with higher overall CMR scores, higher SBP, higher triglyceride levels, and lower HDL cholesterol levels. Being born LGA was associated with higher waist circumference, whereas being born SGA was associated with lower waist circumference. Some data suggested that LGA status may have been associated with higher overall CMR scores, but these results were not statistically significant.

Our results were consistent with several reviews^29,30,31,32,33,34^ that reported associations between preterm birth and CMR. However, those reviews did not find consistent differences in adulthood between those born preterm vs term for all components of the metabolic syndrome, possibly owing to improper adjustment, specifically overadjustment for variables on the causal pathway, such as birth weight. We did not adjust for variables on the causal pathway to ensure that the main exposure estimates were not biased. In a retrospective cohort study of 720 children,^13^ early preterm birth (odds ratio, 3.7; 95% CI, 1.6-8.2) and late preterm birth (odds ratio, 2.5; 95% CI, 1.2-5.3) were associated with metabolic syndrome (ie, central obesity and high levels of triglycerides, HDL cholesterol, blood pressure, and fasting plasma glucose) in adolescence.

Previous studies^10,35,36^ found that both SGA status and LGA status were associated with higher CMR. In a prospective cohort study involving 90 children in Italy, Chiavaroli et al^35^ found that children born LGA and SGA had a higher CMR score compared with children born AGA, with increased differences at adolescence. Kuhle et al^36^ analyzed data from the Canadian Health Measures Survey to find that children born SGA were less likely to have high levels of 3 or more components of metabolic syndrome at ages 6 to 12 years compared with their peers born AGA. A small review by Nordman et al^10^ that investigated the association between size for gestational age and CMR factors reported no differences in fasting glucose levels among children born SGA and LGA compared with those born AGA but did find increased insulin levels and insulin resistance. Both SGA and LGA status were found to be associated with high blood pressure in children and/or adolescents.^10^ However, the review suggested that sex and ethnicity could explain some of the variation among those born LGA.^10^ Overall, there appears to be evidence that the association between size for gestational age and cardiometabolic disease risk may be U-shaped, with children born SGA and LGA having increased risk of some CMR factors, which could be explained by catch-up growth and catch-down growth, respectively, during the first years of life.^10^

These findings may have multiple underlying mechanisms; adverse conditions, both in utero and after birth, occur at a critical stage of organ system development, which could lead to permanent alterations through epigenetic and genetic mechanisms.^37^ The mechanisms associated with hypertension and cardiovascular disorders are believed to involve changes to the vasculature and heart (arterial stiffness and endothelial injury in the absence of circulating progenitor cells),^38^ kidneys (impaired kidney growth leading to a reduction in the number of nephrons, among other theories),^39^ and sympathetic nervous system.^37^ Mechanisms associated with abnormal metabolic homeostasis in those born preterm are complex and remain unclear but could involve changes to the vasculature (arterial stiffness resulting from a low-level state of chronic inflation existing in obesity)^38^ and nutritional deficits both in utero and after delivery.^37^ Catch-up growth in weight without a parallel catch up in length may then result in an obesogenic phenotype similar to that of infants born SGA.^37^ Children born LGA may compensate for higher intrauterine growth through catch-down growth in early years; however, lack of catch-down growth seems to increase the risk of cardiometabolic outcomes in LGA-born young adults.^10^

These findings may have clinical implications. First, they suggest that late preterm birth and possibly LGA status may be important risk factors for cardiometabolic disorders. Because the CMR score tracks risk from childhood into adulthood, early preventive evaluation and CMR monitoring beginning early in childhood is warranted for preterm-born children. To identify these children, medical records and history, including birth history (eg, gestational age, birth weight for gestational age, perinatal complications, and measures of cardiometabolic factors, such as blood pressure), need to be routinely collected for all infants; this information is already regularly collected in clinical practice. In addition, timely treatment options could be developed for children with signs of CMR to reduce subsequent cardiometabolic outcomes.

Advances in neonatal and pediatric care in the past few decades mean that a substantial proportion of children born late preterm are now reaching young to mid-adulthood. Thus, there is a need to understand and prevent the negative cardiovascular health consequences of late preterm birth through targeted interventions before this population reaches older adulthood, when cardiovascular disease incidence increases. Children with a history of preterm birth may need early preventive evaluation and long-term monitoring for cardiometabolic outcomes later in life. Future studies could evaluate whether cardiovascular risk screening for children born preterm improves outcomes among children and young adults and whether population health interventions targeted at children born both early and late preterm mitigate adverse outcomes. More data from larger studies in diverse populations are needed to understand whether recommended perinatal interventions improve preterm birth outcomes and modify the association between late preterm birth and CMR.^37^

### Strengths and Limitations

This study has several strengths. A notable strength was the ability to link clinical data from a large cohort of children to health administrative data from a publicly funded health care system to obtain valid prospectively measured data on gestational age at birth as well as maternal factors and health conditions. Components of the CMR score were measured objectively in childhood by trained staff, and the approach we used to calculate CMR is emerging as a preclinical (or intermediary) cardiometabolic outcome measure among children at risk of developing cardiometabolic disease in adulthood,^40^ allowing for earlier intervention. Extremes of in utero growth, maternal factors, and complications during pregnancy may lead to the decision to induce preterm labor and may also be independently associated with increased CMR later in life. However, a strength of our study was that we were able to control for these potential confounding variables by combining administrative medical records data, cohort study questionnaire data, and physical measures to find that preterm birth was associated with higher CMR in childhood.

The study also has limitations. We cannot rule out the possibility of residual confounding. We have adjusted for height in our SBP models, but national reference standards to allow standardization by age, sex, and height may be necessary for blood pressure measurement in children. Although there were generally few missing variables, missingness for maternal BMI was 14.5%. To address the presence of missing data, multiple imputation was used. Another limitation is that our study was nested within the TARGet Kids! primary care practice–based research network and reflects children recruited from selected primary care practices rather than a population-based representative sample. The findings from this study may not be generalizable to other populations, and selection bias is possible. Children in the study sample were from families that had a relatively high income compared with the overall population.

## Conclusions

In this cohort study, late preterm and moderately preterm birth were associated with higher CMR. Because the CMR score tracks risk from childhood into adulthood, late preterm and moderately preterm birth may be important risk factors for cardiometabolic disorders later in life. Screening and early-life interventions for these children may prevent cardiometabolic outcomes.

## Notes

Supplement. **eTable.** Data Sources Used to Define Study Participant Characteristics and Variable Operationalization **eFigure 1.** Distribution of the CMR *z* Score **eFigure 2.** Directed Acyclic Graph for the Association Between Gestational Age and Cardiometabolic Risk
