# Supplementary material for: Association of Late Preterm Birth and Size for Gestational Age With Cardiometabolic Risk in Childhood
Source: JAMA Netw Open. 2022 May 27;5(5):e2214379. doi: 10.1001/jamanetworkopen.2022.14379 (PMC9142868; doi:10.1001/jamanetworkopen.2022.14379)
Supplement: Supplement. — eTable. Data Sources Used to Define Study Participant Characteristics and Variable Operationalization eFigure 1. Distribution of the CMR z Score eFigure 2. Directed Acyclic Graph for the Association Between Gestational Age and Cardiometabolic Risk [file jamanetwopen-e2214379-s001.pdf]

Supplementary Online Content

Yoshida-Montezuma Y, Sivapathasundaram B, Brown HK, et al. Association of late preterm birth and size for gestational age with cardiometabolic risk in childhood. *JAMA Netw Open*. 2022;5(5):e2214379. doi:10.1001/jamanetworkopen.2022.14379

eTable. Data Sources Used to Define Study Participant Characteristics and Variable Operationalization

eFigure 1. Distribution of the CMR *z* Score

eFigure 2. Directed Acyclic Graph for the Association Between Gestational Age and Cardiometabolic Risk

This supplementary material has been provided by the authors to give readers additional information about their work

© 2022 Yoshida-Montezuma Y et al. *JAMA Network Open*.

eTable. Data Sources Used to Define Study Participant Characteristics and Variable Operationalization

Characteristic	Data Source	Operationalization
**Maternal**		
Age at delivery	MOMBABY	Continuous in years
Pre-pregnancy BMI	BORN + TARGet Kids!	Continuous in kg/m^2
Ethnicity	TARGet Kids!	African/Arab/Latin American/Mixed Ethnicity; East/Southeast/South Asian; and European
Diabetes	ODD	Yes; No
Hypertension	HYPER	Yes; No
**Perinatal**		
Gestational diabetes	BORN	Yes; No
Gestational hypertension	BORN	Yes; No
Preeclampsia/eclampsia	BORN	Yes; No
Venous thromboembolism	OHIP + CIHI-DAD	Yes; No
**Child**		
Gestational age	MOMBABY + BORN	1. Continuous in weeks<br>2. Categorical (<34, 34-36, 37-38, and ≥39 weeks)<br>3. Size for gestational age (<10, 10-90, >90 percentile)
Age at outcome	TARGet Kids!	Continuous in years
Sex	TARGet Kids!	Female; Male
zCMR score	TARGet Kids!	Continuous age and sex standardized z-score of waist circumference, log-triglycerides, glucose, systolic blood pressure, and HDL cholesterol: $(WC + \log(Trig) + Glucose + SysBP - HDL)/\sqrt{5}$
**Sociodemographic**		
Family income	TARGet Kids!	Less than \$49,000; \$50,000 to \$99,999; \$100,000 to \$149,999; \$150,000 or more
Family history of cardiometabolic conditions	TARGet Kids!	Yes; No
BORN: The Better Outcomes Registry & Network, CIHI-DAD: Canadian Institute for Health Information Discharge Abstract Database, HYPER: Ontario Hypertension Database, OHIP: Ontario Health Insurance Plan, ODD: Ontario Diabetes Database		

© 2022 Yoshida-Montezuma Y et al. *JAMA Network Open*.

eFigure 1. Distribution of the CMR *z* Score

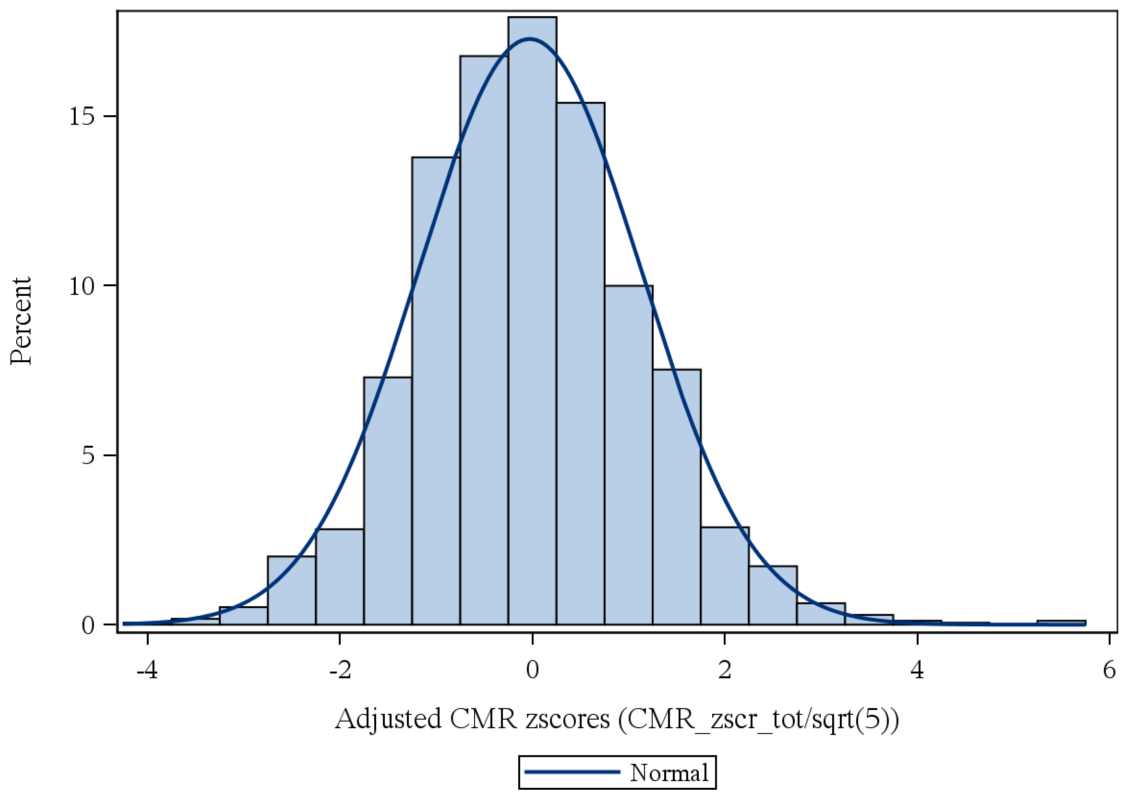

© 2022 Yoshida-Montezuma Y et al. *JAMA Network Open*.

eFigure 2. Directed Acyclic Graph for the Association Between Gestational Age and Cardiometabolic Risk

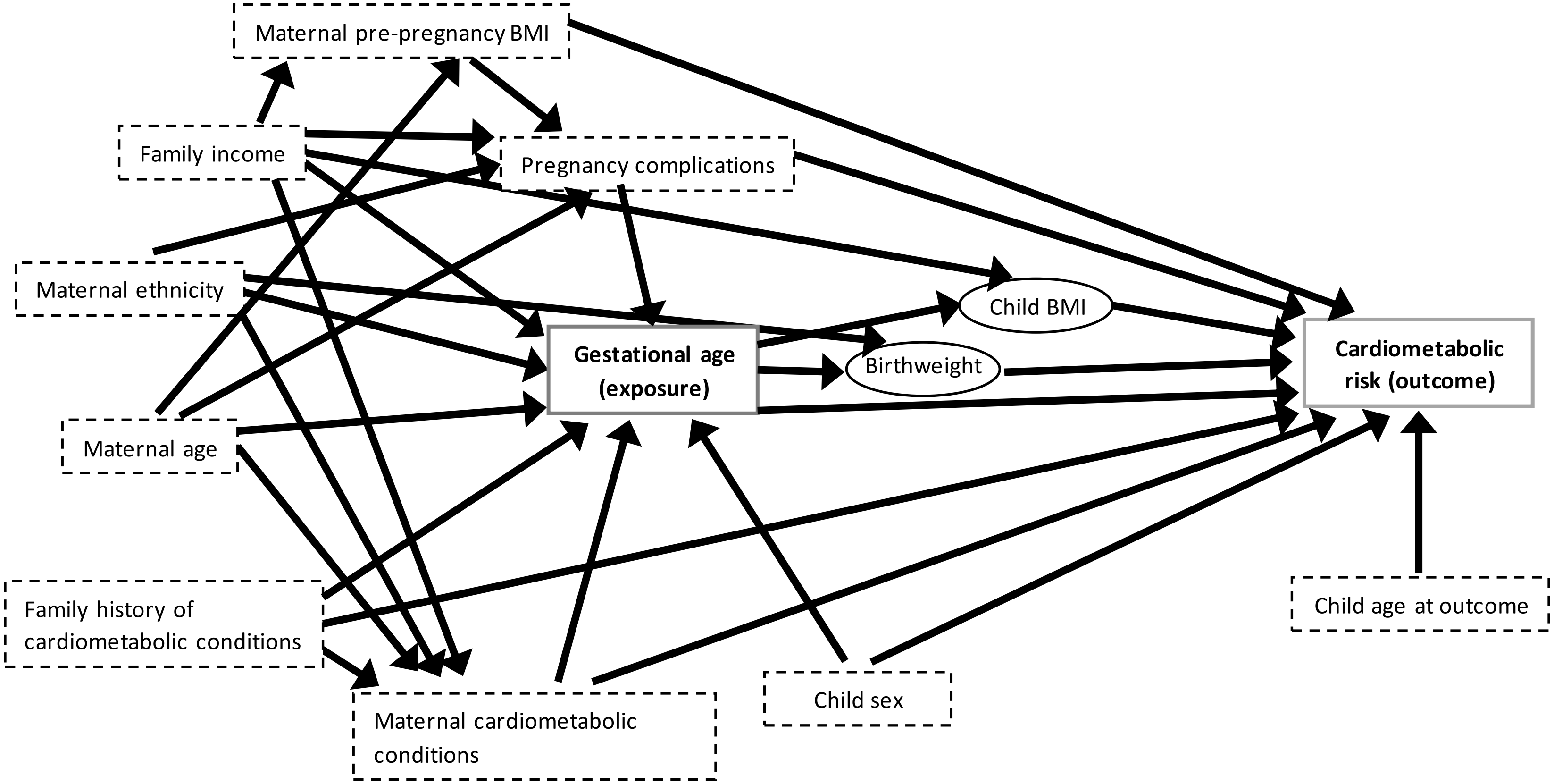

Directed acyclic graph (DAG) for the relationship between gestational age and cardiometabolic risk identifying the minimum sufficient set of potential confounders in dashed square boxes and potential mediators (which were not conditioned on) in circles. Unobserved variables were not included.

© 2022 Yoshida-Montezuma Y et al. *JAMA Network Open*.
